# Impact of measurement timing on reproducibility of testing among haemodialysis patients

**DOI:** 10.1038/s41598-021-02526-2

**Published:** 2022-01-19

**Authors:** Anna Junqué Jiménez, Ester Tomás Bernabeu, Lola Andreu Périz, Eva Segura Ortí

**Affiliations:** 1 Nephrology Department, Hospital de Terrassa, Consorci Sanitari de Terrassa, Crta Torrebonica s/n, 08227 Terrassa, Spain; 2 Nursing Department, Facultat de Medicina i Ciències de la Salut, Universitat de Barcelona, Barcelona, Spain; 3 Physiotherapy Department, Universidad Cardenal Herrera-CEU, CEU Universities, Valencia, Spain

**Keywords:** Health care, Nephrology

## Abstract

Accurate evaluation of physical function in patients undergoing haemodialysis is crucial in the analysis of the impact of exercise programs in this population. The aim of this study was to evaluate the reproducibility of several physical functional tests, depending on the timing of their implementation (before the HD session vs. non-HD days). This is a prospective, non-experimental, descriptive study. Thirty patients in haemodialysis were evaluated twice, 1 week apart. The test session was performed before the haemodialysis session started and a retest was performed in non-dialysis day. The testing battery included the short physical performance battery, sit-to-stand tests, 6 min walk test, one-leg stand test, timed up and go, and handgrip strength with and without forearm support. The intra-rater reproducibility was determined by the intraclass correlation coefficients and the agreement was assessed by Bland–Altman analysis. The intraclass correlation coefficients values ranged from 0.86 to 0.96, so that all tests showed good to very good relative reliability. The mean differences between trials of sit to stand 10 and 60, timed up and go and all the handgrip tests were close to zero, indicating no systematic differences between trials. Large range of values between trials was observed for the 6 min walk test, gait speed, one-leg stand test and short physical performance battery, indicating a systematic bias for these four tests. In conclusion,  the sit to stand 10 and 60, timed up and go and handgrip tests had good to excellent test–retest reliability in measuring physical function in different dialysis days of patients undertaking haemodialysis. The minimal detectable change values are provided for this population. Bias were found for the 6 min walk test, gait speed, Short physical performance battery or one-leg stand test when the testing day changed.

## Introduction

Chronic kidney disease (CKD) is an important global health problem because of its high incidence, prevalence, morbidity and mortality rates, and socioeconomic cost^1,2^. Globally, an estimated 850 million people suffer from kidney disease, amounting to more than 10% of the adult population and accounting for at least 2.4 million deaths annually^3^.

Haemodialysis (HD) is the most common form the renal replacement therapy (RRT). Patients on long-term HD experience physical function problems, as well as an impaired health-related quality of life^4^.

Physical activity (PA) is any body movement produced by muscles that results in increased energy expenditure. Exercise is a subset of PA that is planned, structured, repetitive and purposeful^5^. PA level is lower in CKD patients at any stage compared to healthy counterparts^6,7^. It seems that PA level has also an impact on physical function since patients in HD or peritoneal dialysis with impaired PA had worst physical function compared with more active patients^8,9^. Several studies have recently been published which report the beneficial effects of exercise on the physical function of patients receiving HD^4,10–12^. Physical function tests are commonly used to assess the effectiveness of exercise programs and may be challenging for patients and their assessors to complete due to time constrains before the start of the HD session.

Previous studies have investigated the relative and absolute reliability of several physical functional tests, many of which have demonstrated excellent test–retest intra-rater reliability when tests are undertaken before the start of the HD session^13–17^. Reproducibility refers to the variation in measurements made on a subject under changing conditions^18^. It remains unknown if physical functional tests are reproducible when the same rater measures in non-HD days.

Therefore, the aim of this study was to evaluate the reproducibility of several physical functional tests, depending on the timing of their implementation (before the HD session vs. non-HD days).

## Materials and methods

### Design

This was a reproducibility and method comparison study, changing day of testing, HD versus non-HD day.

### Setting and participants

The participants were recruited from the HD unit in the Hospital de Terrassa in August 2019 and signed a written informed consent. This study was approved by the Ethics Committee at Hospital de Terrassa and was carried out in accordance with the standards set out in the Declaration of Helsinki, it was registered at ClinicalTrials.gov (NCT04049708), Patients were included in the study if they had been receiving maintenance HD for at least 3 months and did not have any acute or chronic medical conditions that would preclude collection of the test data. Individuals were excluded if they had recently had a myocardial infarction (in the 6 weeks prior), or had unstable angina, malignant arrhythmias, or any disorder that would be exacerbated by activity. Demographic and clinical data from the patients’ medical histories were registered.

### Procedure

The study consisted of repeating the same tests in two different occasions, trials 1 and 2, to evaluate the reproducibility. It was always performed by the same experienced nurse. The test session was performed before HD treatment, as described elsewhere in the literature^17,19,20^. Before the first HD session of the week, the participants underwent the short physical performance battery (SPPB), one-leg stance test (OLST), and timed up and go (TUG) tests. Before the second HD session in the same week, the patients performed the Sit to stand 10 (STS-10) and sit to stand 60 (STS-60) tests. Finally, the participants undertook the 6 min walk test (6MWT) before their third HD session in the week.

The retest session was performed on a non-HD day by the same nurse. Participants completed the same battery of tests in a single test session.

### Definition the tests

#### Short physical performance battery (SPPB)

Objectively measures lower extremity function and includes several tests, balance, gait speed, and sit to stand 5 repetitions (STS-5). This is a commonly used test in patients undertaking HD^17,21^.

#### One-leg standing test (OLST)

It consists of maintaining a one-leg stance for as long as possible, with a maximum of 45 s per leg in three trials^19^.

#### Timed up-and-go test (TUG)

The participants were given verbal instructions to stand up from a standard armchair (using their arms if necessary), walk 3 m as quickly and safely as possible, turn back at a cone set out by the researchers, walk back, and sit down again in the chair. The patients could wear their regular footwear and to use a walking aid if needed. A stopwatch was started on the word “go” and stopped when the patient was fully seated again with their back against the backrest. The time taken to complete the test was recorded in three consecutive trials, using the first one to familiarise the patients with the test. The best time from the three trials was analysed^22^.

#### Sit-to-stand tests (STS)

The STS10 consisted of performing 10 complete movements of sitting down and standing as fast as possible, with the arm held tightly against the chest. STS10 elapsed time was recorded. In the STS-60 test, the number of repetitions performed for 60 s was recorded^17,20,23^.

#### Handgrip (HG) with or without arm support

Two different procedures were compared, with and without arm support. In the HG test without support, the participant was seated in a chair. Participants performed three consecutive 3 s repetitions using an approved Jamar hand dynamometer, with 15 s rest periods between repetitions. The same test was then performed with the arm supported by the surface of a table providing support^24,25^.

#### The 6-min walk test (6MWT)

It consisted of assessing the maximum distance walked during a 6 min period^26^.

### Statistics

The normality of the data distribution was assessed using the Kolmogorov–Smirnov test. Normally distributed descriptive data were reported as the mean plus the standard deviations (*SDs*) and non-parametric data were reported as the median plus the range. We also performed paired comparisons with paired *t*-tests or Wilcoxon signed rank tests to assess any systematic bias between the trials.

Bland–Altman plots were used to visually assess the disagreement between the measurements in two different measurement days. A plot of each participant’s mean score plotted against the patient score difference (test on non-dialysis day minus retest before HD treatment) was constructed to check for possible systematic bias. The Bland–Altman plots displayed the 95% limits of agreement (95% LOA) which give a range within which it is expected the 95% of future differences in measurements between measurement days to lie. The 95%LOA was calculated as the difference in the mean scores of the test ± the score difference *SD* × 1.96.

The intraclass correlation coefficient (ICC; model alpha) and a two-way random-effects model were used to assess relative intra-rater reliability which was rated ‘excellent’ (ICC ≥ 0.900), ‘good’ (≥ 0.750) or ‘fair’ (0.600 to 0.749)^27^. We assumed that there was no systematic bias between measurements within subjects and that the within-subject SDs were equal for all measurements since the same rater measured participants 1-week apart.

We calculated the absolute reliability, standard error of measurement (SEM), and minimal detectable change (MDC) 90% confidence interval (MDC_90_) thresholds for these tests. The SEM and the MDC_90_ were calculated using the following formulas^17,23^.

$${\text{SEM}} = SD \times \sqrt {\left( {1 - r} \right)} ,$$

where *r* = ICC for the participant group and MDC_90_ = SEM × 1.65 × $\sqrt{2}.$

The SEM measures absolute reliability and represents the extent to which a variable can fluctuate during the measurement process^28^.To be 90% confident about the range for a measurement, the calculation 1.68 × SEM was used^15,16^. The MDC is defined as the amount of change in a measurement required to conclude that the difference is not attributable to error and is the smallest change that falls outside the expected range of error^16,29,30^. We set the level of significance required to a probability of *p* ≤ 0.05 for all our statistical analyses and the data were managed and analysed using the Statistical Package for the Social Sciences (SPSS) version 20.0 for windows (IBM Corp., Armonk, NY).

## Results

Thirty participants with a mean age of 66.4 years (*SD* = 16.3), mean time on HD of 34.4 months (*SD* = 51.4), and mean Charlson comorbidity index of 8.5 (*SD* = 2.5) completed this study. The demographic and clinical data statistics for all the participants are shown in Table 1. No adverse events occurred during the testing.

**Table 1:** Demographic, biochemical, haematological, and dialysis adequacy data as well as nutritional parameters for the patient cohort.

**Demographic data**	
Age (years) ${\overline{\text{X}}}$ (*SD*)	66.4 (16.3)
Time on HD (months) ${\overline{\text{X}}}$ (*SD*)	34.4 (51.4)
Sex (% men)	66.7
Charlson index ${\overline{\text{X}}}$ (*SD*)	8.5 (2.5)
Glomerular disease (%)	13.3
Hypertension (%)	13.3
Diabetes mellitus (%)	23.3
**Biochemical data** ${\overline{\mathbf{X}}}$ **(*SD*)**	
Glucose (mg/dl)	133.6 (55.7)
Creatinine (mg/dl)	8.2 (3.1)
k (mEq/l)	5.5 (0.6)
Ca (mg/dl)	9.2 (0.7)
P (mg/dl)	4.7 (1.4)
i-PTH (pg/ml)	499.5 (664.2)
25-OH Vitamin D (ng/ml)	23.9 (9.6)
**Nutritional parameters** ${\overline{\mathbf{X}}}$ **(*SD*)**	
Albumin (g/dl)	3.8 (0.4)
Total cholesterol(mg/dl)	153.2 (47.2)
HDL cholesterol (mg/dl)	42.2 (14.9)
LDL cholesterol (mg/dl)	78.4 (29.3)
Triglycerides (mg/dl)	160.3 (135.1)
Objective nutritional assessment	26.53 (4.1)
**Haematological data** ${\overline{\mathbf{X}}}$ **(*SD*)**	
Haemoglobin (g/dl)	11.5 (1.3)
Ferritin (ng/ml)	378.6 (198.7)
**Dialysis adequacy** ${\overline{\mathbf{X}}}$ **(*SD*)**	
Dialysis dose (Kt/v)	1.8 (0.6)

*N* = 30.

*Ca* calcium, *HD* haemodialysis, *HDL* high-density lipoprotein, *i-PTH* intact parathyroid hormone, *k* potassium, *kt/v* Daugirdas formula for second-generation logarithmic estimates of single-pool variable volume, *LDL* low-density lipoprotein, *P* phosphorus.

Descriptive statistics of trial 1 (before the HD session) and trial 2 (non-dialysis day) as well as differences, are shown in Table 2.

**Table 2:** Descriptive values (N = 30).

	Trial 1 Before the HD session Median (Min–Max) Mean (SD)	Trial 2 Non-HD day Median (Min–Max) Mean (SD)	Trial 2–1 Difference Median (Min–Max) Mean (SD)	Range
SPPB (points)	11 (7–12)	11 (7–12)	0 (− 1–1)	2
4-m gait speed (m/s)	1.1 (0.6–2.0)	1.2 (0.6–2.2)	− 0.09 (0.1)	0.58
STS-10 (s)	25.2 (12.2–49.5)	25.6 (13–70)	0.66 (− 13.3–22.0)	35.3
STS-60 (repetitions)	22 (11–45)	22.5 (9–42)	0 (− 9.0–6.0)	15
6MWT (m)	436.5 (262–735)	425 (214–721)	− 15.7 (39.5)	150
OLST (s)	4.3 (0–45)	6.5 (0–45)	0 (− 7.0–28.3)	35.3
TUG (s)	8.9 (4.6–22)	9.1 (4.6–20)	0.2 (1.3)	5.7
HG with support R (kg)	22 (12–54)	23 (11–49)	1.1 (4.0)	13
HG with support L (kg)	20.5 (6–46)	20.5 (10–49)	1.1 (3.3)	18
HG without support R (kg)	21.5 (10–53)	22 (10–49)	0.7 (3.0)	13
HG without support L (kg)	19.5 (8–48)	20 (10–50)	0.6 (2.7)	12

*n* = 30.

*HD* haemodialysis, *HG* handgrip strength, *L* left side, *OLST* one-legged stance test, *R* right side, *SPPB* short physical performance battery, *STS10* sit-to-stand 10 test, *STS60* sit-to-stand 60 test, *TUG* timed up-and-go test, *6MWT* 6-min walking test.

Overall, MDC and SEM were quite large, especially for the 6MWT. Since SEM values can be translated to normal curve probabilities, Table 3 values can be applied to the practice. Using STS-10 as the example, it can be expected with the probability of 96% chance that the value of repeated tests will be in approximately ± 7.2 s of the original value.

**Table 3:** Values of intra-rater relative and absolute reliability (haemodialysis day vs. non-haemodialysis day).

	ICC (95% CI)	MDC_90_	*SEM*
SPPB	0.947 (0.891–0.974)	0.9	0.4
4-m gait speed (m/s)	0.863 (0.733–0.933)	0.3	0.1
STS-10 (s)	0.861 (0.729–0.931)	8.5	3.6
STS-60 (repetitions)	0.925 (0.848–0.963)	5.4	2.3
6MWT (m)	0.932 (0.861–0.963)	68.8	29.5
OLST (s)	0.896 (0.794–0.949)	14.1	6.1
TUG (s)	0.945 (0.887–0.973)	2.1	0.9
HG with support R (kg)	0.945 (0.887–0.973)	5.5	2.3
HG with support L (kg)	0.910 (0.921–0.956)	6.8	2.9
HG without support R (kg)	0.955 (0.908–0.978)	5.1	2.2
HG without support L (kg)	0.950 (0.899–0.976)	4.6	1.2

*n* = 30.

*CI* confidence interval, *HD* haemodialysis, *HG* handgrip strength, *ICC* intraclass correlation coefficient, *L* left side, *MDC*_*90*_ minimal detectible change score 90% confidence interval, *OLST* one-legged stance test, *R* right side, *SEM* standard error mean, *SPPB* short physical performance battery, *STS10* sit-to-stand 10 test, *STS60* sit-to-stand 60 test, *TUG* timed up-and-go test, *6MWT* 6-min walking test.

Given the value of the MDC calculated in the present study is 8., and the value of the test in both trials is around 25 s, these results suggest that a change in the individual performance of less than one third of the mean cannot be considered a real change and it would be considered a measurement error for the STS-10.

Intraclass correlation coefficients values ranged from 0.86 to 0.96, so that all tests showed good to very good relative reliability (Table 3). Confidence intervals were narrow, except for the relatively large confidence interval obtained for gait speed test and the STS-10.

Bland–Altman scatterplots were created to estimate disagreement between the two trials. The mean differences of STS-10, STS-60, TUG and all the handgrip tests were close to zero, indicating no systematic differences between trials. All, except for the handgrip tests, presented better values on non HD day.

**Figure 1:**
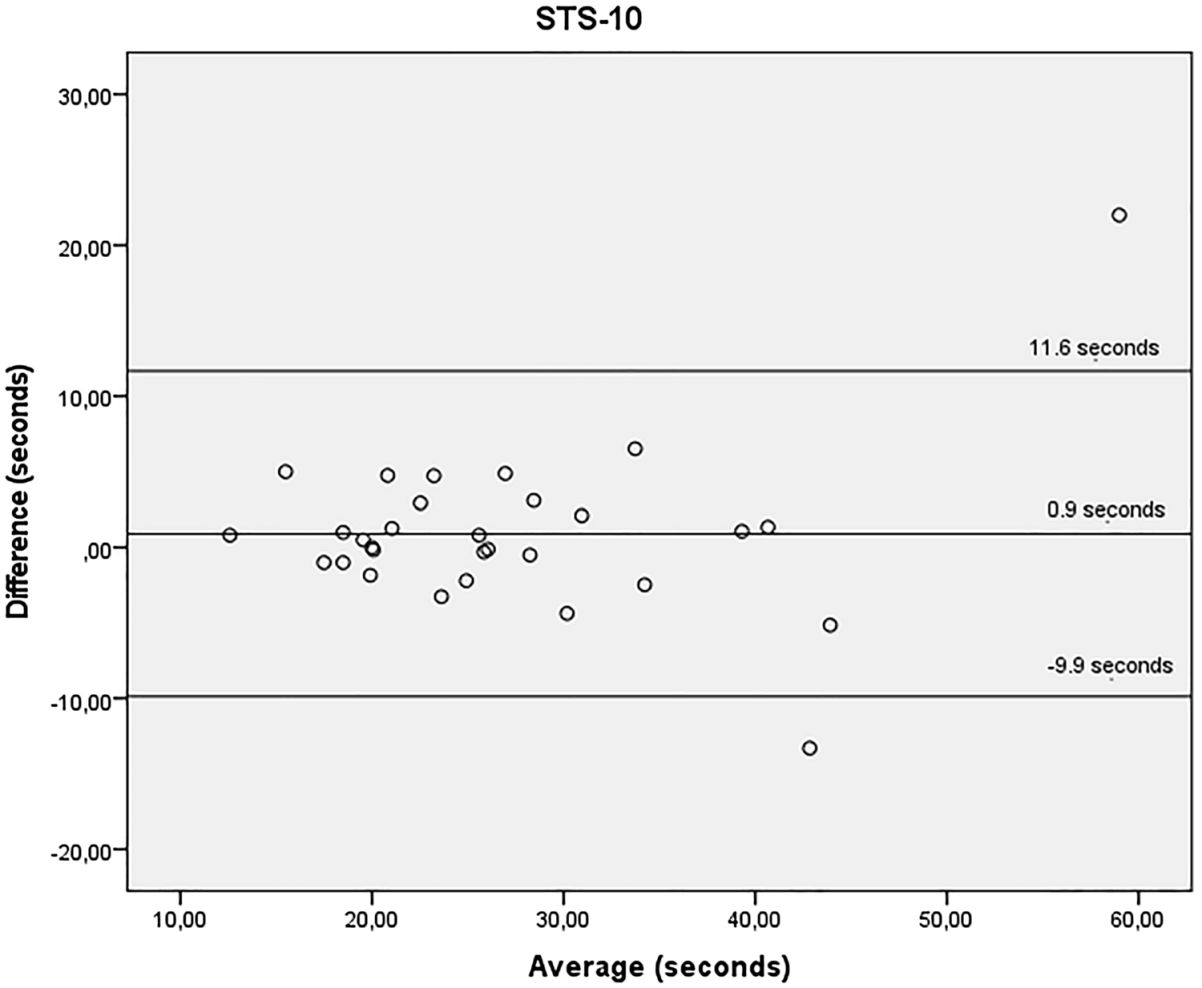
Bland–Altman plots showing agreement for the time required to perform the sit-to-stand-to-sit 10 test, obtained before the haemodialysis session and on a non-dialysis day by the same rater. Y axis difference between (non-dialysis—before the haemodialysis session) in seconds. X axis average (non-dialysis + before the haemodialysis session)/2 s.

**Figure 2:**
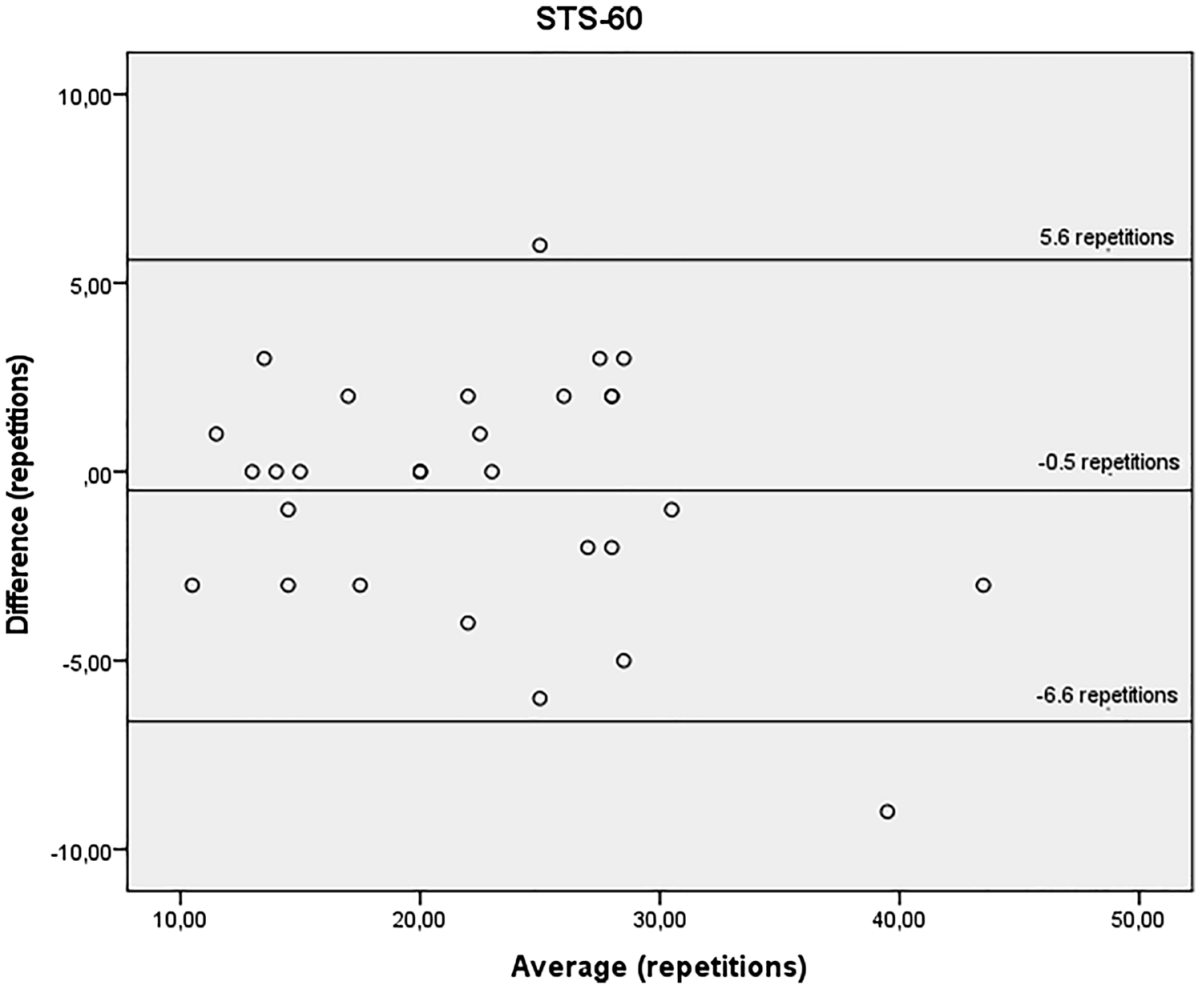
Bland–Altman plots showing agreement for the time required to perform the sit-to-stand-to-sit 60 test, obtained before the haemodialysis session and on a non-dialysis day by the same rater. Y axis difference between (non-dialysis—before the haemodialysis session) in seconds. X axis average (non-dialysis + before the haemodialysis session)/2 s.

**Figure 3:**
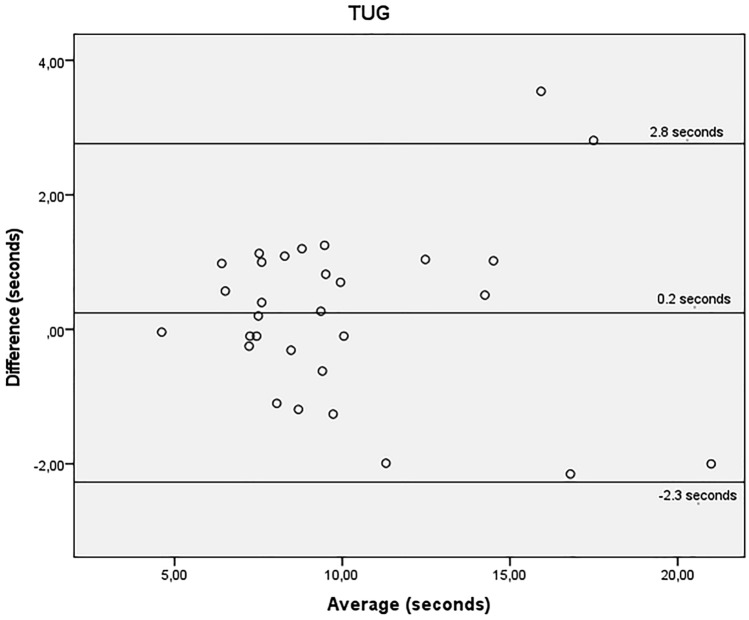
Bland–Altman plots showing agreement for the time required to perform the timed up-and-go test, obtained before the haemodialysis session and on a non-dialysis day by the same rater. Y axis difference between (non-dialysis—before the haemodialysis session) seconds. X axis average (non-dialysis + before the haemodialysis session)/2 s.

Figures 1, 2 and 3 show the agreement between STS-10 (Fig. 1), STS-60 (Fig. 2), and TUG (Fig. 3) before the HD session and on a non-dialysis day. For the STS-10 there was a mean difference of 0.9 s between the days (95% LOA − 9.9 and 11.6 s). For the STS-60 there was a mean difference of − 0.5 repetitions (95% LOA − 6.6 and 5.6 repetitions). For the TUG there was a mean difference of 0.2 s (95% LOA − 2.3 and 2.8 s).

**Figure 4:**
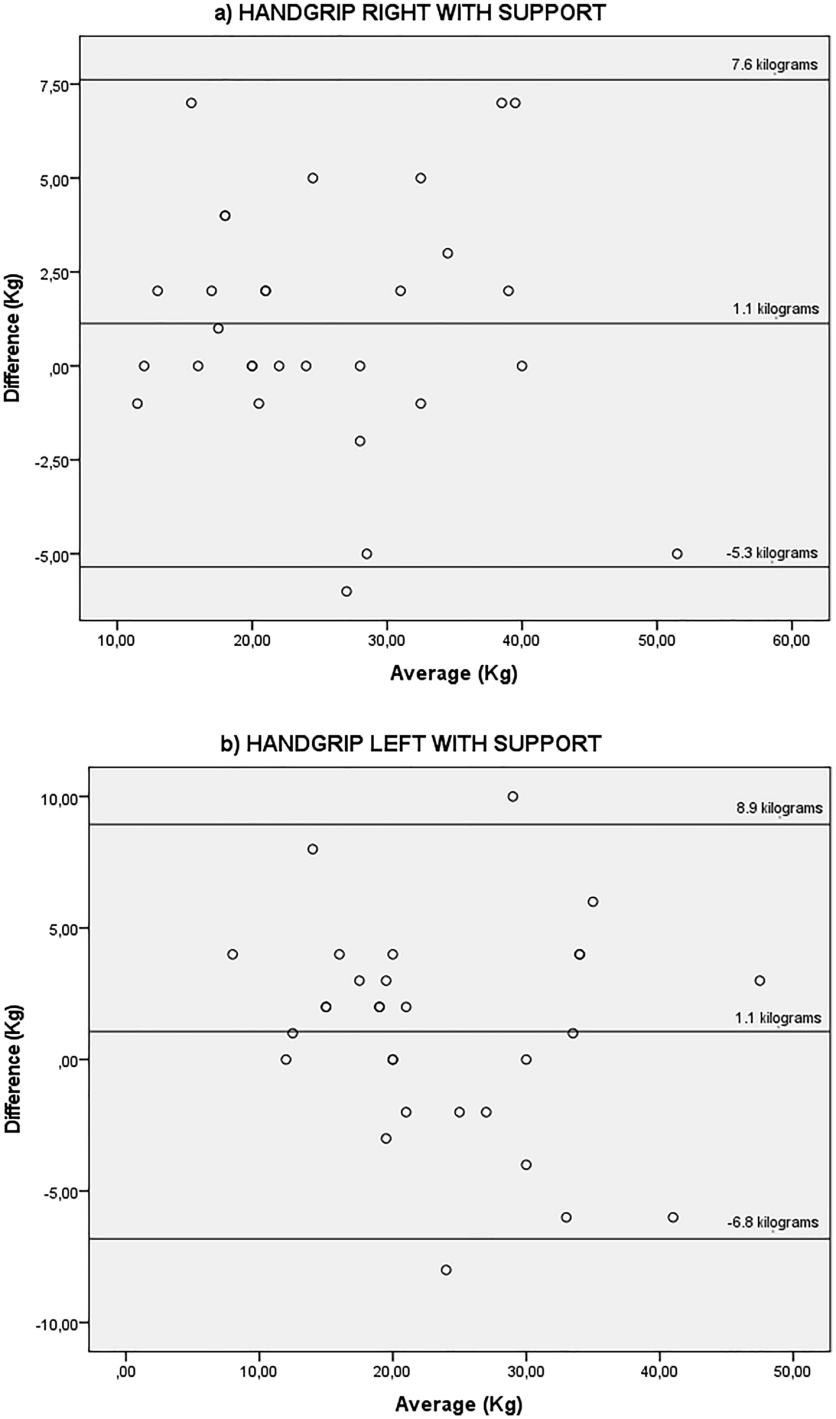
Bland–Altman plots showing agreement for the kilograms achieved with the handgrip strength test, right and left with forearm supported, obtained before the haemodialysis session and on a non-dialysis day by the same rater. Y axis difference between (non-dialysis—before the haemodialysis session) Kilograms X axis average (non-dialysis + before the haemodialysis session)/2 kg.

**Figure 5:**
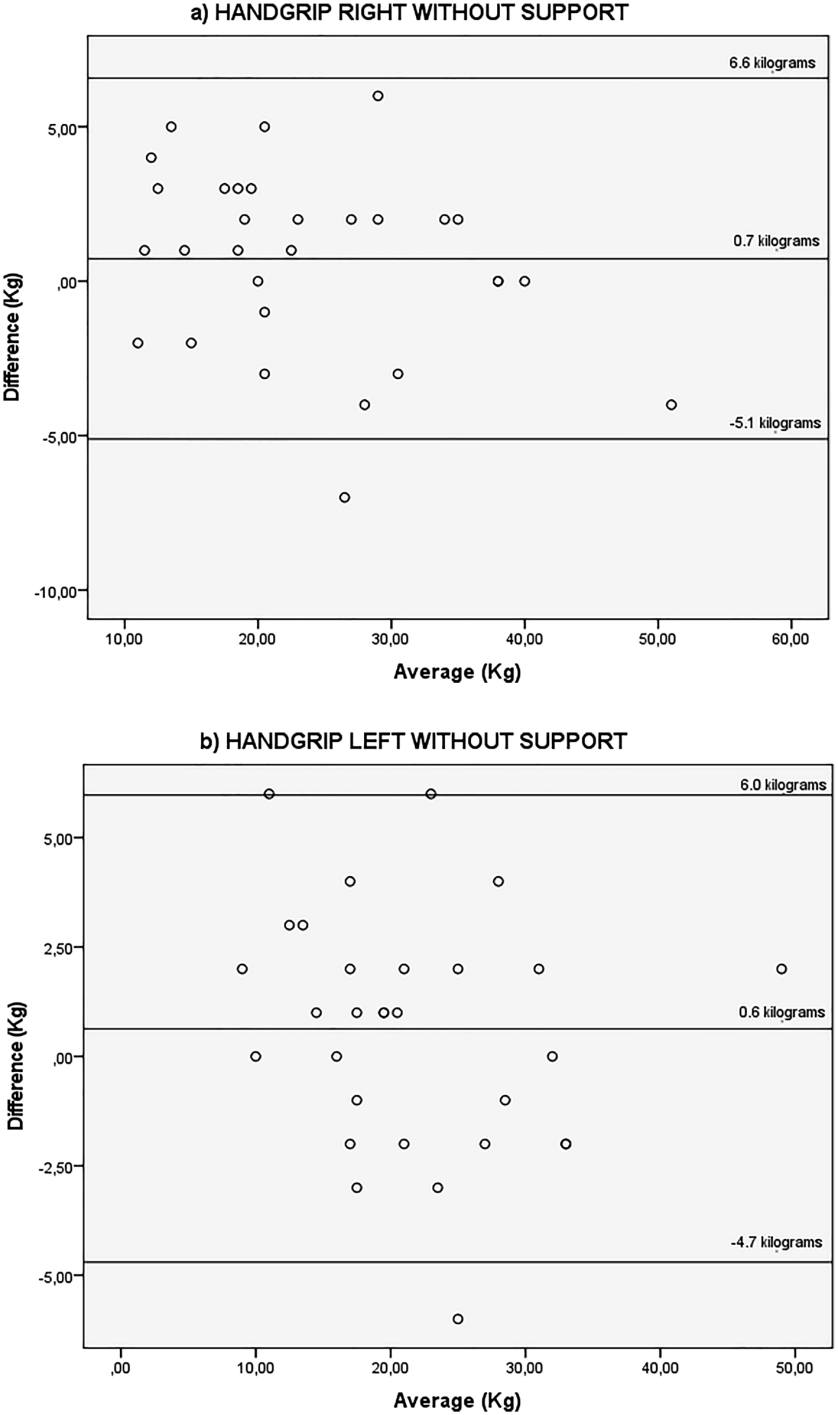
Bland–Altman plots showing agreement for the kilograms achieved with the handgrip strength test, right and left without support, obtained before the haemodialysis session and on a non-dialysis day by the same rater. Y axis difference between (non-dialysis—before the haemodialysis session) Kilograms X axis average (non-dialysis + before the haemodialysis session)/2 kg.

Figures 4 and 5 show the agreement between HG strength test with support Fig. 4 right hand and left hand, and without forearm support Fig. 5 right hand and left hand before the HD session and on a non-dialysis day. For the HG strength with forearm support there was a mean difference of 1.1 kg between the days for the right (95% LOA − 5.3 and 7.6 kg) and 1.1 kg for the left hand (95% LOA − 6.8 and 8.9 kg). For the HG strength without forearm support there was a mean difference of 0.7 kg between the days for the right hand (95% LOA − 5.1 and 6.6 kg) and 0.6 kg for the left hand (95% LOA − 4.7 and 6.0 kg).

All figures show that there is not much change in the differences as the mean increased while the variation of data was constant.

**Figure 6:**
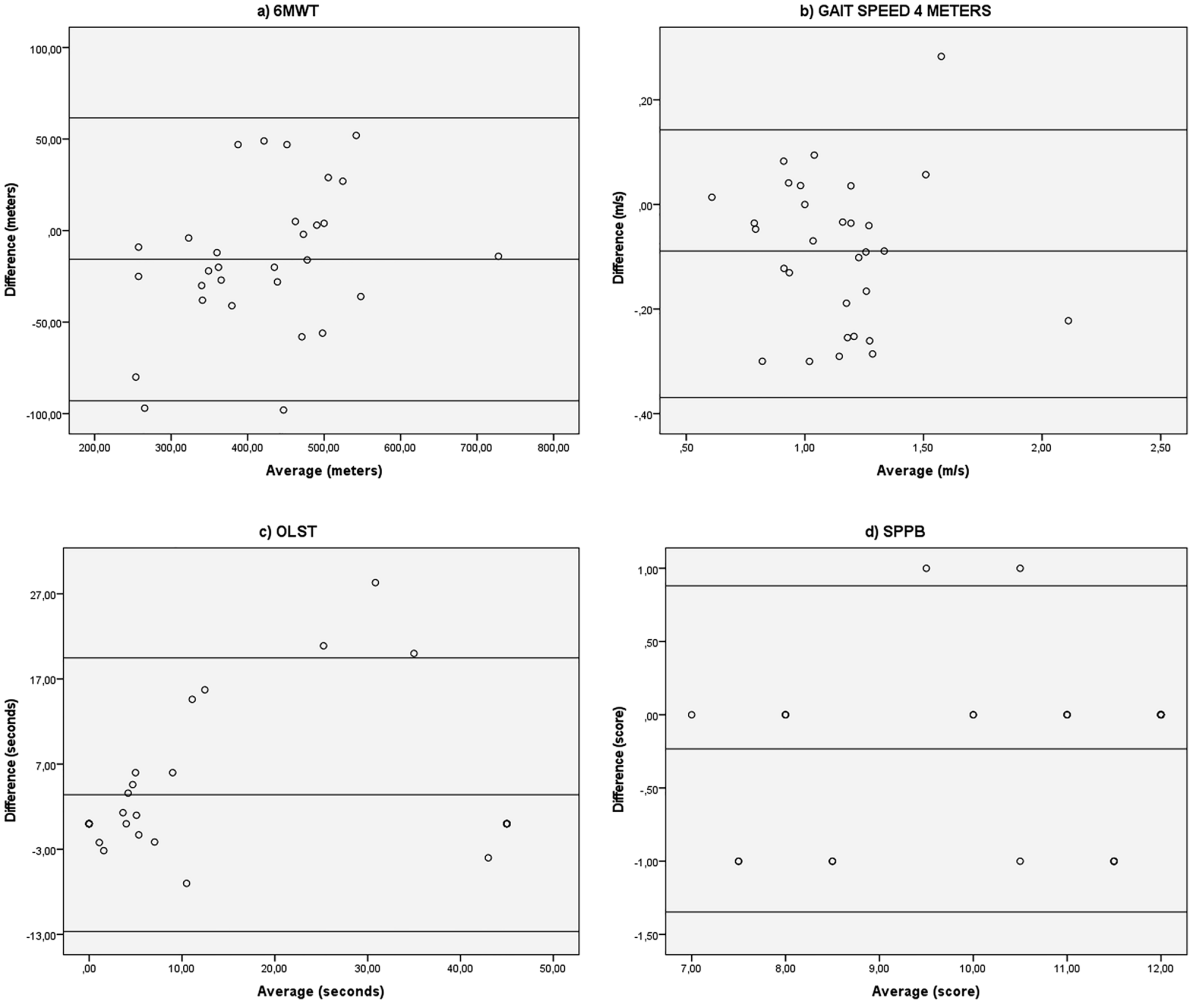
Bland–Altman plots for the tests that showed systematic bias: 6 min walk test; gait speed in 4 m; one-leg stand test; short physical performance battery.

Large range of values between trials was observed for the 6MWT, gait speed, OLST and SPPB (Table 2). Thus, Bland–Altman plots indicated a systematic bias for these four tests (Fig. 6). The mean difference scores between the different days for the same rater differed significantly from exact agreement (*p* < 0.001).

## Discussion

The study attempted to clarify if physical function tests measured in patients undertaking HD are reproducible when changing the testing day (before the HD session vs. non-dialysis day). The sample size reached the recommended number of 30^31^.

Although high ICC coefficients were obtained, ICC is a ratio index of within and between subjects’ variability, therefore agreement between groups of subjects does not provide information about the individual change or error in scores. Additionally, ICC is dependent of the sample variability, and thus ICC should not be employed isolated^32^. The Bland–Altman plots were useful in exposing the relationship between the trials.

The present study shows a high degree of agreement between measurements on different days (HD day before the session vs. non-HD days) and good or excellent ICC results (above 0.86) only for some tests (STS-10, STS-60, TUG and HG tests) demonstrating lack of systematic bias when the measurement day changed. Thus, our results support the use of these tests when there is a change in the timing for assessment.

The scores from our participants were the similar to those reported by previous research of our group, with a slight difference only for the handgrip tests (STS-10: 25.2–25.6 s vs. 25.1–24.0 s; STS-60: 22–22.5 repetitions vs. 25.6–25.5 repetitions; TUG: 8.9–9.1 s vs. 9.0–8.6 s; HG right: 22–23 kg vs. 26.9–25.9 kg; HG left: 20.5–20.5 kg vs. 23.8–23.4 kg)^19,23^. Our sample was around 5 years older than the previous samples studied. Compared to other studies, with HD patients around 62 and 57 years old, results are also similar, for the STS-60, with 26–28 repetitions^23^, and 20.5–19.8 repetitions^33^, this last article differing from the rest, probably due to the small sample of only 10 patients. For the TUG, it is reported 8.9–8.1 s^33^.

Our results suggest that without arm support HG test is also reliable and has even lower values of MDC, what would made it easier to find true changes out of the variability of the measurement.

The present ICC results concur with those from our previous studies in similar samples (39 participants for the STS-10, STS-60, HG)^17^ or in larger samples (71 participants for the TUG)^19^ (STS10: 0.861 vs. 0.88; STS60: 0.925 vs. 0.97; TUG: 0.945 vs. 0.96; HG right 0.945 vs. 0.96; HG left 0.925 vs. 0.95). They are also in agreement with values reported by other studies for STS-60 (0.927)^23^, Our ICC values are better compared to the values of a small study with 10 patients (STS-60: 0.84; TUG 0.71)^33^. However, to the best of our knowledge this is the first work to check agreement and reproducibility when the timing of the test administration (before the HD session vs. non-HD day) is changed.

The SEM and MDC_90_ found in the current study, compared to previous studies are similar for the SEM (STS10: 3.6 vs. 3.6 s; STS60: 2.3 vs. 1.7 repetitions; TUG: 0.9 vs. 1.24 s; HG right 2.3 vs. 1.5 kg; HG left 2.9 vs. 1.5 kg) and for the MDC_90_ values (STS10: 8.5 vs. 8.4 s; STS60: 5.4 vs. 4 repetitions; TUG: 2.1 vs. 2.9 s; HG right 5.5 vs. 3.4 kg; HG left 6.8 vs. 3.4 kg). In general, apart from the TUG, measurement variation is higher when measures are taken in different days, before HD and non-dialysis days, so these data support the recommendation of avoiding changing the testing day to decrease absolute reliability values. These data are in agreement with previous data, (STS-60: SEM values 1.3^23^–2.43^33^ repetitions; MDC_95_, 4 repetitions^23^, MDC_90_ 5.47 repetitions^33^).

Our results show that there was no systematic bias for the STS-10, STS-60, TUG, or HG tests and so, these tests can be measured on different days. Nevertheless, this study shows a systematic bias for the SPPB, gait speed, and 6MWT when the timing (before the HD session vs. non-dialysis day) changes. Systematic bias have been explained by the learning effect once the participant repeats the test and improves results during the re-test, albeit to a non-significant degree^34^. A previous intra-rater study also showed a non-learning effect^19^. Our results do not show this learning effect, since gait speed and 6MWT performance was better before the HD session on trial 1 compared to the retest session on non-HD days (Table 2). Some authors suggest that the testing before the HD session may have reduced the effects of fatigue from the previous HD session^33^. Additionally, it is well-known the high variability of functional results in this cohort^17,20^, so it seems very important to keep the same testing circumstances when testing this cohort.

Hence, the use of Bland–Altman method evidenced that 6MWT, gait speed, OLST and SPPB showed substantial bias and large disproportion of the LOA. This case, large ICC values but lack of agreement with Bland–Altman method, was also found when establishing reliability of some motor tests^32^. Gait speed, and 6MWT achieved higher results when testing before the HD session, while balance achieved higher results on non-HD days. Fatigue, as a result of administering all the tests in a row on a non-HD day could explain why some tests obtained poorer results on non-HD days, which should not affect balance. Previous research has tested a battery of three test on non-HD days^33^. Clinical feasibility does not allow us to test patients on several non-HD days because these participants already spend many hours in a clinical setting for their treatments and so it would be difficult to convince them to spend extra time in for physical function testing alone. Finally, our results may help to clarify which tests could be measured before the HD session by the same rater, because there is no consensus on this regard and clinical applicability should be considered to extend testing into routine treatment.

The main strength of this study was that, to the best of our knowledge, this was the first time that the reproducibility of physical function tests in patients undergoing HD has been tested with different test administration timings. Assessment at the nephrology units could be difficult to implement because of a lack of human resources and logistics in many clinical settings. Thus it is important to be flexible regarding the test timing in this cohort, but it is also important to note that these changes impact the reproducibility of several commonly used physical function tests. The main weaknesses of this work were that the sample size was relatively small. Another limitation is that we did not make two measurements with each timing. Since there was only 1-week difference between measurements, we believe we may assume that there were no systematic biases between measurements within subjects and that the within-subject SDs were similar for all measurements.

Our results have important implications in the implementation of physical function testing in HD units and indicate that the same assessors should test patients. Future work should be multicentric and include higher sample sizes to confirm it and should also aim to clarify the ideal battery for clinical assessments in this population by assessing other tests, such as lower-muscle strength tests.

## Conclusion

The STS-10, STS-60, TUG and handgrip tests had good to excellent test–retest reliability in measuring physical function in different dialysis days of patients undertaking HD. The MDC values are provided for this population. Bias were found for the 6MWT, gait speed, SPPB, or OLST when the testing day changed. Future studies should be conducted to clarify the ideal battery for routine clinical assessments in this population, including lower-limbs muscle strength tests.
